# Reliability and validity assessment of the Chinese version of the Intrahospital Transport Safety Scale (IHTSS) in intensive care units

**DOI:** 10.1186/s12912-024-01906-z

**Published:** 2024-04-29

**Authors:** Shuaishuai Li, Shuting Hou, Xianjiao Deng, Shihao Chen, Huaqin Wang, Li Tang, Man Ye, Jianhui Xie

**Affiliations:** 1 Clinical Nursing Teaching and Research Section, The Second Xiangya Hospital of Central South University, No. 139 Renmin Middle Road, 410011 Changsha, Hunan China; 2 Critical Care Medicine, The Second Xiangya Hospital of Central South University, 410011 Changsha, Hunan China; 3 Department of Nursing, The Affiliated Children’s Hospital of Xiangya School of Medicine, Central South University (Hunan children’s hospital), No. 86 Ziyuan Road, 410011 Changsha, Hunan China

**Keywords:** Intrahospital transport, Critically ill patients, Patient safety, Critical care, Reliability, Validity, Scale

## Abstract

**Background:**

Intrahospital transport of critically ill patients is a common practice in intensive care units (ICUs), where patients’ safety is constantly challenged in high-intensity and dynamic environments. While Intrahospital Transport Safety Scale (IHTSS) is widely used internationally to evaluate the intrahospital transport safety, it has not been introduced in China.

**Objectives:**

This study aimed to assess the reliability and validity of the Chinese version of the IHTSS scale among critical care nurses in China.

**Methods:**

A cross-sectional study was conducted using a cluster sampling method. A total of 544 critical care nurses from 25 ICUs in 10 tertiary hospitals were recruited. We employed exploratory factor analysis (EFA) and confirmatory factor analysis (CFA) to examine the questionnaire’s underlying factor structure, ensuring construct validity. Additionally, internal consistency was assessed using Cronbach’s alpha coefficient, test-retest reliability, and corrected item-total correlation.

**Results:**

The Chinese version of the scale displayed robust psychometric properties, with a Cronbach’s α coefficient of 0.976, a split-half reliability of 0.906, and a test-retest reliability of 0.856. EFA revealed a robust four-factor model that accounted for 75.970% of the variance, with the factor loadings of the items ranging from 0.433 to 0.951. CFA indicated a strong model fit, with a chi-square to degrees of freedom ratio (CMIN/DF) of 2.765, comparative fit index (CFI) of 0.943, incremental fit index (IFI) of 0.943, and goodness-of-fit index (GFI) of 0.845, supporting the efficacy of the four-factor model in assessing intrahospital transport safety for critically ill patients.

**Conclusion:**

The Chinese version of the IHTSS demonstrated favourable reliability and validity among critical care nurses in China, making it a suitable tool for measuring the level of intrahospital transport safety for critically ill patients.

## Background

Patient safety is a paramount concern in healthcare, with the World Health Organization designating “Patient Engagement and the Maintenance of Patient Safety” as the theme for World Patient Safety Day 2023 [1–3]. Among all the medical units, the intensive care unit (ICU) offers the highest level of life support, offering meticulous monitoring and care to patients [4]. However, the demanding workload of ICU nurses, complex patient conditions, and dynamic environment place ICU patients at a heightened risk for adverse events [5–7]. These risks are particularly elevated during intrahospital transport, when patients are exposed to a mobile and ever-changing environment with limited resources [8].

Fragile ICU patients often require transportation between different hospital departments for examinations and treatments, which compromises their safety. Firstly, physical factors such as temperature fluctuations, acceleration, and navigating uphill and downhill slopes can destabilize a patient’s hemodynamics during movement. Secondly, emergencies that arise during transport present more uncertainties due to the limited resources available in external environments compared to the well-equipped ICU ward [9]. Furthermore, patient safety during intrahospital transport is influenced by various factors, including the implementation of specific safety measures and policies within the hospital [10]. These measures may include standardized transport protocols, checklists, and training programs for healthcare professionals involved in patient transport [11]. Healthcare professionals receive training through simulation exercises, hands-on clinical practice, classroom instruction, and continuous education programs to enhance their skills and knowledge in patient transport safety [12]. Additionally, regular reviews and updates of training materials help ensure that healthcare professionals stay informed about the latest best practices and guidelines. As such, ensuring patient safety during intrahospital transport of critically ill patients is an issue that demands significant attention within the context of the ICU.

The purpose of these transfers is to provide patients with additional medical diagnosis and treatment options [13]. In terms of team collaboration, effective communication and coordination among healthcare professionals were essential during ICU patient transportation within the hospital [8]. Physician typically assessed patient stability, determined the need for additional diagnostic or therapeutic interventions, and ensured the availability of suitable transportation equipment and personnel when issuing transport orders [14]. While some hospitals had established standards for transport orders, the specific protocols and guidelines varied depending on the institution [13]. It was essential for hospitals to have clear and standardized procedures for intrahospital patient transport to ensure patient safety and minimize adverse events. Once a transport order is received, critical care nurses will be responsible for implementing intrahospital transport, including preparing the patient for transport, monitoring their condition during the transfer, and managing their care after the transfer [15]. Numerous studies have highlighted the high frequency of intrahospital transfers among critically ill patients, with approximately 43.5% of ICU patients requiring such transfers [16–18]. However, it is concerning to note that the global incidence rate of adverse events during these transfers is 26.2%, while in China, it is as high as 79.8% [19, 20]. These adverse events commonly involve complications related to the respiratory system, cardiovascular issues, neurological concerns, and problems with equipment [8, 21–23]. Consequently, patient safety is consistently compromised during these transfers.

To mitigate adverse events during intrahospital transports and ensure the safety of patients, researchers have developed various tools, one of which is the pre-transport checklist [24, 25]. Additionally, Professor Lina [26] has introduced the IHTSS to effectively measure the safety of intrahospital transport for critically ill patients, this scale has proven to be instrumental in understanding safety prerequisites and enhancing clinical practice. By utilizing a self-report approach, IHTSS evaluates intrahospital transport safety from five dimensions: organization, tools and technology, environment, teamwork, and transport-related tasks. This comprehensive evaluation provides valuable insights into the safety aspects of intrahospital transport. Notably, the scale has demonstrated favourable reliability and validity within the specific context of intensive care in Sweden, further highlighting its efficacy as a tool for assessing the safety of intrahospital transport.

There are limited approaches to address safety concerns related to intrahospital transport of critically ill patients from the perspective of human factors engineering (HFA) in China. Possible reasons are as follows. Firstly, the existing assessment tools in China lack consistency. The emergency department graded transport model is used for the intrahospital transport of critically ill patients in the emergency department, aiming to ensure safety during the transport process [27]. Others used Healthcare Failure Mode and Effect Analysis (HFMEA) to improve the safety of intrahospital transport of critically ill patients [12]. However, there is inconsistency among ICU nurses regarding the protocols and checklists for intrahospital transport of critically ill patients [28]. This lack of consistency means that the quality and safety of intrahospital transport cannot be compared across various ICUs. Secondly, critical care nurses play a crucial role in facilitating intrahospital transport, actively participating in all stages of the process [29]. Historically, there has been a paucity of adverse events reported by nurses concerning intrahospital transport [30]. Therefore, there is a need to introduce a comprehensive assessment tool that can evaluate the safety and quality of intrahospital transport for critically ill patients.

The IHTSS encompasses multiple dimensions of transport safety and is utilized through a self-report scale by critical care nurses. Introducing the IHTSS within the context of ICU healthcare in China holds significant potential applications. This scale aims to increase awareness and attention to the quality of intrahospital transport, evaluating the safety of intrahospital transport for critically ill patients from multiple dimensions and perspectives. Therefore, there is an urgent need to introduce the IHTSS to ensure timely treatment and safe transport of critically ill patients to relevant collaborative departments, ultimately improving clinical outcomes. Hence, the objective of this study is to investigate the reliability and validity of the IHTSS among critical care nurses in China.

## Methods

### Design and sample

This study was conducted from June to October 2022 among critical care nurses working in the ICUs of tertiary hospitals throughout Hunan Province, China. A cross-sectional design and a cluster sampling approach were employed to gather data. The first step involved randomly selecting 10 hospitals from 50 tertiary hospitals in Hunan province using a simple random method. These 10 hospitals were located in the cities of Changsha, Hengyang, Shaoyang, and Xiangtan. In the second step, all adult ICU units in each hospital were included, and critical care nurses were recruited.

We opted for cluster sampling as it provided an efficient means to select critical care nurses from a range of hospitals in Hunan Province. This approach entailed the random selection of entire hospitals as clusters, facilitating the recruitment of nurses from various ICUs within each hospital. By doing so, we were able to capture a diverse array of healthcare environments, thereby enhancing the overall representativeness of our study.

To be eligible, nurses needed to have been working in the ICU and participated in intrahospital transport of critically ill patients within the past year. Nurses currently on rotation, undergoing training, pursuing further education or internships, as well as those who had been on leave for more than three months, were excluded from the study. To ensure the accuracy and reliability of the study results, the research adhered to the criterion proposed by Kendall [31], which suggests a minimum of 10 respondents per item. As the scale used in this study contained 24 items, the minimum required sample size was calculated to be 240 participants. Considering a potential 20% rate of invalid questionnaires, the researchers aimed to collect at least 300 valid samples. The survey successfully obtained participation from a total of 761 critical care nurses working in 25 ICUs across the 10 selected hospitals.

### Instrument

The IHTSS [26] is a comprehensive tool designed to assess the safety level of intrahospital transport for critically ill patients. It consists of 24 items that are categorized into five dimensions: organization (6 items), tools and technology (5 items), environment (5 items), teamwork (4 items), and transport-related tasks (4 items) The Likert 5-point rating scale was employed, ranging from “strongly disagree” to “strongly agree”. The total score range for the scale is 24 to 120, with a higher score indicating a higher level of intrahospital transport safety. The original scale demonstrated good internal consistency, with a Cronbach’s α coefficient of 0.88. The subscales also showed acceptable levels of internal consistency, with Cronbach’s α coefficients ranging from 0.72 to 0.82.

### Procedures

### Translation procedure

The IHTSS was translated into Simplified Chinese following the Brislin model [29, 30] by the researchers to ensure the accuracy and cultural adaptability of the scale. The translation process consisted of three steps.

In Step 1, two Chinese nursing professionals who were proficient in both English and Chinese independently translated the original scale into Chinese using a forward-backward translation procedure. The first author then compared and merged the two translations and engaged in discussions with experts to resolve any discrepancies. This iterative process resulted in the final version of the translated scale.

In Step 2, two teachers from the School of Foreign Languages, who were proficient in English but not familiar with the original scale, conducted a back-translation. The back-translation was performed separately by both teachers, and the resulting versions were then compared and discussed in order to come up with a consensus. The final translated scale was found to be consistent with the original version.

In Step 3, an expert panel was formed, consisting of three researchers, including a psychologist and two nursing experts. The panel compared the translated and back-translated versions with the original scale, considering cultural adaptation and ensuring equivalence of idiomatic concepts to align the language expressions with mainland Chinese linguistic norms.

Finally, a convenience sample of 30 critical care nurses from a tertiary hospital in Changsha, China was selected to participate in a pilot experiment. After completing the scale, all participants expressed that the scale had a clear theme, a well-structured format, coherent logic, and clear semantics. The feedback from the pilot participants further supported the clarity and usability of the translated scale.

### Data collection procedure

This study adopted a cluster sampling design to collect data. Specifically, 10 tertiary hospitals in 4 cities in Hunan province were selected as the sample. To conduct the survey, an online platform called Wenjuan Star was utilized. Once the sampling results were obtained, the survey questionnaire links were distributed to the directors of the nursing departments, who subsequently shared the links with the nurses working in the ICUs. The questionnaire was designed in a standardized format and language, and participants were required to provide electronic informed consent before proceeding. Each item in the questionnaire was mandatory. Any abnormal questionnaires that exhibited obvious regularity or confusing logic, such as having the same choices for answers or contradictory responses, were excluded from the data analysis. In total, 761 critical care nurses participated in the survey. Out of these, 544 valid questionnaires were collected, resulting in an effective recovery rate of 71.5%. To assess the retest reliability of the scale, a second survey was conducted with a random selection of 30 nurses two weeks later.

### Data analysis

SPSS 25.0 and AMOS 24.0 were employed for statistical analysis. All tests were two-tailed and the significance level was set at two-sided α = 0.05. Descriptive statistics, comprising mean and standard deviation for continuous variables, as well as frequencies and percentages for demographic factors, were calculated by SPSS. The item analysis of the scale adopts the item distribution method, critical ratio method and Spearman correlation coefficient method. Reliability assessment included Cronbach’s alpha coefficient, split-half reliability, and a two-week test-retest intraclass correlation coefficient (ICC). Validity was assessed through content validity index (CVI), EFA and CFA. The sample of 544 participants was randomly divided into two groups: one group (*n* = 262) for EFA and the other group (*n* = 282) for CFA. CFA was conducted using AMOS software to analysis the goodness of fit indices of the model. EFA utilized principal component analysis and direct oblimin rotation with maximum variance in SPSS. Item analysis, reliability assessment, and CVI were also analyzed using SPSS.

### Ethics approval

The study protocol was approved by the Medical Ethics Review Committee of the University of South China (approval number: xy-2021-39). All methods employed in this study adhered to the principles outlined in the Declaration of Helsinki. Before the commencement of the study, electronic written informed consent was obtained from each participant.

## Results

### The sample

Among the 544 study participants, their ages ranged from 20 to 50 years, with a mean age of 31.39 ± 5.32 years. There were 75 male participants (13.8%) and 469 female participants (86.2%). On average, they had been working in critical care for 6.95 ± 5.16 years. The detailed demographic data was shown in Table 1. The total mean was 94.91, SD = 13.23 and the average scores for each dimension and each item were shown in Table 2.

**Table 1:** Frequency distribution of demographic characteristics (*n* = 544)

Variables	Groups	N	%
Sex	Male	75	13.8
	Female	469	86.2
Age(years)	$$ <$$25	62	11.4
	25 ∼ 30	168	30.9
	31 ∼ 40	293	53.8
	41 ∼ 50	21	3.9
Work years	$$ <$$1	32	5.9
	1 ∼ 5	205	37.7
	6 ∼ 10	186	34.2
	11 ∼ 20	143	21.3
	$\geq$20	5	0.9
Education level	College	19	3.5
	Undergraduate	469	86.2
	Postgraduate	56	10.3
Job title	Nurse	42	7.7
	Senior Nurse	171	31.4
	Supervisor Nurse	322	59.2
	Co-Chief Nurse	9	1.7
Type of intensive care unit	RICU*	53	9.7
	General ICU*	174	32.0
	CCU*	27	5.0
	EICU*	13	2.4
	NICU*	75	13.9
	SICU*	143	21.0

* RCIU: Respiratory care unit; General ICU: General intensive care unit; CCU: Coronary Care Unit; EICU: Emergency Intensive Care Unit; NICU: Neurosurgical intensive care unit; SICU: Surgical intensive care unit

**Table 2:** Mean (SD) scores and skewness and kurtosis values of the scale (*n* = 544)

Item	Mean $\pm$SD	Skewness	Kurtosis
Organization	22.41$\pm$4.34	-0.32	0.41
Q1	3.62$\pm$0.91	-0.41	-0.18
Q2	3.67$\pm$0.86	-0.46	0.18
Q3	3.96$\pm$0.70	-0.53	1.19
Q4	3.73$\pm$0.82	-0.46	0.22
Q5	3.77$\pm$0.80	-0.48	0.40
Q6	3.66$\pm$0.89	-0.62	0.29
Tools And Technology	20.54$\pm$2.74	-0.02	0.13
Q7	4.02$\pm$0.66	-0.33	0.33
Q8	4.08$\pm$0.64	-0.24	0.08
Q9	4.13$\pm$0.62	-0.46	1.34
Q10	4.15$\pm$0.63	-0.31	0.17
Q11	4.17$\pm$0.58	-0.19	0.61
Environment	19.54$\pm$3.17	-0.15	0.41
Q12	3.83$\pm$0.84	-0.73	0.74
Q13	3.95$\pm$0.72	-0.56	0.88
Q14	3.73$\pm$0.86	-0.54	0.10
Q15	3.88$\pm$0.77	-0.53	0.42
Q16	4.15$\pm$0.62	-0.20	-0.05
Team Work	16.02$\pm$2.40	-0.06	0.33
Q17	3.91$\pm$0.75	-0.58	0.81
Q18	4.06$\pm$0.63	-0.36	0.96
Q19	4.09$\pm$0.60	-0.24	0.52
Q20	3.97$\pm$0.72	-0.67	1.20
Transport-Related Tasks	16.41$\pm$2.25	-0.06	0.52
Q21	4.08$\pm$0.64	-0.78	2.83
Q22	4.12$\pm$0.60	-0.30	0.82
Q23	4.10$\pm$0.59	-0.35	1.10
Q24	4.10$\pm$0.59	-0.24	0.70

### Item analysis

In this study, based on Pearson correlation analysis, there was a statistically significant strong correlation between the items and the total score (*P* < 0.001), with correlation coefficients ranging from 0.694 to 0.805 (Table 3). The critical ratio for the 24 items in this study ranged from 26.083 to 32.413 (Table 4), indicating good discriminative ability of the items. All items were positively correlated with the total score, showing a moderate to high level of correlation. The Cronbach’s α values of the scale remained between 0.968 and 0.970 after removing each item, which did not exceed the overall Cronbach’s α value of the scale (0.970).

**Table 3:** IHTSS item-total score person correlation analysis results (*n* = 544, α = 0.05)

Item	Item content	$\varvec{r}$	*p*
Q1	We had sufficient staff resources to prepare for the transport.	0.759	$$ <$$0.001
Q2	We had enough time to prepare for the IHT.	0.761	$$ <$$0.001
Q3	IHT preparation in the ICU was well coordinated.	0.771	$$ <$$0.001
Q4	We had sufficient staff resources to settle the patient back in the ICU.	0.762	$$ <$$0.001
Q5	We had enough time to settle the patient back in the ICU.	0.776	$$ <$$0.001
Q6	I was able to perform IHT-related tasks without being interrupted.	0.668	$$ <$$0.001
Q7	The transport equipment met the requirements needed to perform the transport safely.	0.774	$$ <$$0.001
Q8	The transport equipment was reliable.	0.774	$$ <$$0.001
Q9	It was easy to monitor the patient throughout the IHT.	0.770	$$ <$$0.001
Q10	Audible alarms supported my work in monitoring the patient.	0.717	$$ <$$0.001
Q11	Medical tools (IV lines, tubes, cords and so on) were suited to the intended purpose.	0.743	$$ <$$0.001
Q12	The physical layout of the hospital facilitated safe performance of the transport.	0.703	$$ <$$0.001
Q13	The physical layout of the ICU facilitated preparation for the transport.	0.805	$$ <$$0.001
Q14	Rooms at the destination sites were designed for ICU patients.	0.694	$$ <$$0.001
Q15	Hallways were free from obstacles.	0.752	$$ <$$0.001
Q16	We were able to maintain the patient’s privacy during the transport.	0.700	$$ <$$0.001
Q17	A team leader was clearly recognised.	0.755	$$ <$$0.001
Q18	We gave each other feedback throughout the transport.	0.797	$$ <$$0.001
Q19	We confirmed each other’s responsibilities.	0.779	$$ <$$0.001
Q20	All team members were present when transfer information was shared.	0.789	$$ <$$0.001
Q21	Individual team members knew what tasks they had to perform.	0.794	$$ <$$0.001
Q22	The skills of staff on our IHT team overlapped sufficiently so that work could be shared when necessary.	0.767	$$ <$$0.001
Q23	We had a shared understanding of the task sequence during the IHT.	0.791	$$ <$$0.001
Q24	I felt supported by the other team members.	0.766	$$ <$$0.001

**Table 4:** Discriminant validity analysis of the Chinese version of the scale (*n* = 544)

Item	Low-score group (mean$\pm$SD)	High-score group (mean$\pm$SD)	*t*	*p*
Q1	4.51 ± 0.71	2.70 ± 0.68	27.193	$$ <$$0.001
Q2	4.53 ± 0.68	2.82 ± 0.68	26.334	$$ <$$0.001
Q3	4.70 ± 0.49	3.14 ± 0.60	29.632	$$ <$$0.001
Q4	4.57 ± 0.62	2.89 ± 0.63	28.054	$$ <$$0.001
Q5	4.57 ± 0.60	2.93 ± 0.63	27.762	$$ <$$0.001
Q6	4.44 ± 0.79	2.78 ± 0.72	22.817	$$ <$$0.001
Q7	4.74 ± 0.46	3.20 ± 0.60	30.435	$$ <$$0.001
Q8	4.79 ± 0.41	3.28 ± 0.59	31.266	$$ <$$0.001
Q9	4.82 ± 0.41	3.35 ± 0.63	28.898	$$ <$$0.001
Q10	4.87 ± 0.37	3.41 ± 0.62	30.071	$$ <$$0.001
Q11	4.87 ± 0.34	3.46 ± 0.59	30.814	$$ <$$0.001
Q12	4.61 ± 0.66	2.94 ± 0.71	25.381	$$ <$$0.001
Q13	4.75 ± 0.48	3.13 ± 0.62	30.383	$$ <$$0.001
Q14	4.59 ± 0.69	2.92 ± 0.64	26.083	$$ <$$0.001
Q15	4.71 ± 0.54	3.08 ± 0.65	28.171	$$ <$$0.001
Q16	4.84 ± 0.39	3.41 ± 0.65	27.901	$$ <$$0.001
Q17	4.73 ± 0.53	3.13 ± 0.65	28.100	$$ <$$0.001
Q18	4.82 ± 0.39	3.29 ± 0.58	32.413	$$ <$$0.001
Q19	4.83 ± 0.37	3.34 ± 0.58	32.135	$$ <$$0.001
Q20	4.76 ± 0.46	3.17 ± 0.61	31.006	$$ <$$0.001
Q21	4.82 ± 0.39	3.29 ± 0.68	29.146	$$ <$$0.001
Q22	4.84 ± 0.36	3.38 ± 0.65	29.254	$$ <$$0.001
Q23	4.82 ± 0.39	3.36 ± 0.61	30.026	$$ <$$0.001
Q24	4.83 ± 0.71	3.38 ± 0.61	29.591	$$ <$$0.001

### Validity analysis

#### Content validity

The Chinese version of IHTSS was assessed by a panel of 24 experts. The evaluation results revealed an I-CVI score range of 0.88 to 1.00 and an S-CVI value of 0.83.

### Construct validity

#### Exploratory factor analysis

The 544 questionnaires were randomly divided into two equal groups using SPSS software for further analysis. One half was used for conducting EFA, while the other half was used for CFA. Out of the total sample,262 questionnaires were employed for EFA. The EFA results revealed a Kaiser-Meyer-Olkin(KMO) value of 0.949 and a significant Bartlett’s test of sphericity value of 6099.242(*P* < 0.001), indicating a strong intercorrelation among the variables and suitability for factor analysis [32]. Through principal component analysis and direct oblique rotation, four factors with eigenvalues greater than 1 were extracted, explaining a cumulative variance of 75.970%. The component matrix, after oblique rotation, demonstrated that all item loadings were above 0.4, ranging from 0.433 to 0.951. Please refer to Table 5 for detailed results.

**Table 5:** Factor load and communalities of each item in IHTSS of 24 Items (*n* = 262)

Item	F1	F2	F3	F4	Communalities
24	**0.951**	-0.045	0.017	-0.017	0.867
22	**0.942**	0.066	-0.016	-0.083	0.856
23	**0.926**	-0.003	0.018	-0.024	0.852
21	**0.907**	0.086	-0.056	-0.003	0.844
19	**0.769**	-0.075	0.133	0.107	0.771
20	**0.599**	0.267	0.031	0.101	0.726
18	**0.562**	-0.029	0.223	0.227	0.718
17	**0.503**	0.094	-0.059	0.234	0.703
2	-0.026	**0.906**	0.056	-0.031	0.820
5	0.004	**0.876**	0.051	-0.002	0.816
1	0.053	**0.853**	-0.060	0.082	0.803
4	0.015	**0.818**	-0.003	0.085	0.758
6	-0.027	**0.651**	0.062	0.259	0.690
3	0.175	**0.621**	0.204	-0.043	0.692
9	-0.012	0.082	**0.849**	0.031	0.807
11	0.065	0.087	**0.812**	-0.054	0.765
10	-0.017	-0.076	**0.763**	0.174	0.651
8	0.132	0.216	**0.703**	-0.089	0.766
7	0.186	0.342	**0.515**	-0.068	0.704
16	0.397	-0.153	**0.433**	0.214	0.635
14	0.085	0.241	-0.130	**0.730**	0.749
15	0.145	0.076	0.072	**0.724**	0795
12	-0.056	0.081	0.291	**0.653**	0.705
13	0.125	0.137	0.300	**0.497**	0.739

The results revealed that all 24 items had loadings greater than 0.4, suggesting that all of them should be retained. The component matrix showed that Factor 1 included items 17, 18, 19, 20, 21, 22, 23, and 24, while Factor 2 consisted of items 1, 2, 3, 4, 5, and 6. Factor 3 comprised items 7, 8, 9, 10, and 11, and Factor 4 included items 12, 13, 14, 15, and 16. The scree plot indicated a flattening slope after the fifth factor, suggesting that no additional significant factors were present. Therefore, retaining four factors were considered appropriate. The results also demonstrated some variations in the dimensional structure and individual item attribution between the Chinese version of IHTSS and the original scale. For detailed results, please refer to Table 6.

**Table 6:** Item comparison table after exploratory factor analysis

Dimensions	Item	Factor	Item
Organisation	1, 2, 3, 4, 5, 6	Factor 2	1, 2, 3, 4, 5, 6
Tools and technologies	7, 8, 9, 10, 11	Factor 3	7, 8, 9, 10, 11, **16**
Environment	12, 13, 14, 15, 16	Factor 4	12, 13, 14, 15
Teamwork	17, 18, 19, 20	Factor 1	17, 18, 19, 20 21, 22, 23, 24
Transport-related task	21, 22, 23, 24		

According to Table 6, notable changes in the dimensional structure of the scale have emerged. Specifically, all items originally associated with the Teamwork and Transport-related task dimensions in the original scale have been consolidated into a single dimension in the Chinese version. Furthermore, Item 16, previously categorized under the Environment dimension, has been relocated to the Tools and Technology dimension. After careful consideration of the content of Item 16, it was concluded that the exposure of patient privacy due to inappropriate use of nursing tools could be attributed to cultural differences. Therefore, placing Item 16 under the Tools and Technology dimension was deemed acceptable. After extensive deliberations among the research team, it was agreed to rename the Teamwork and Transport-related task dimensions as Transport-related Tasks and Collaboration.

### Confirmatory factor analysis

**Fig. 1:**
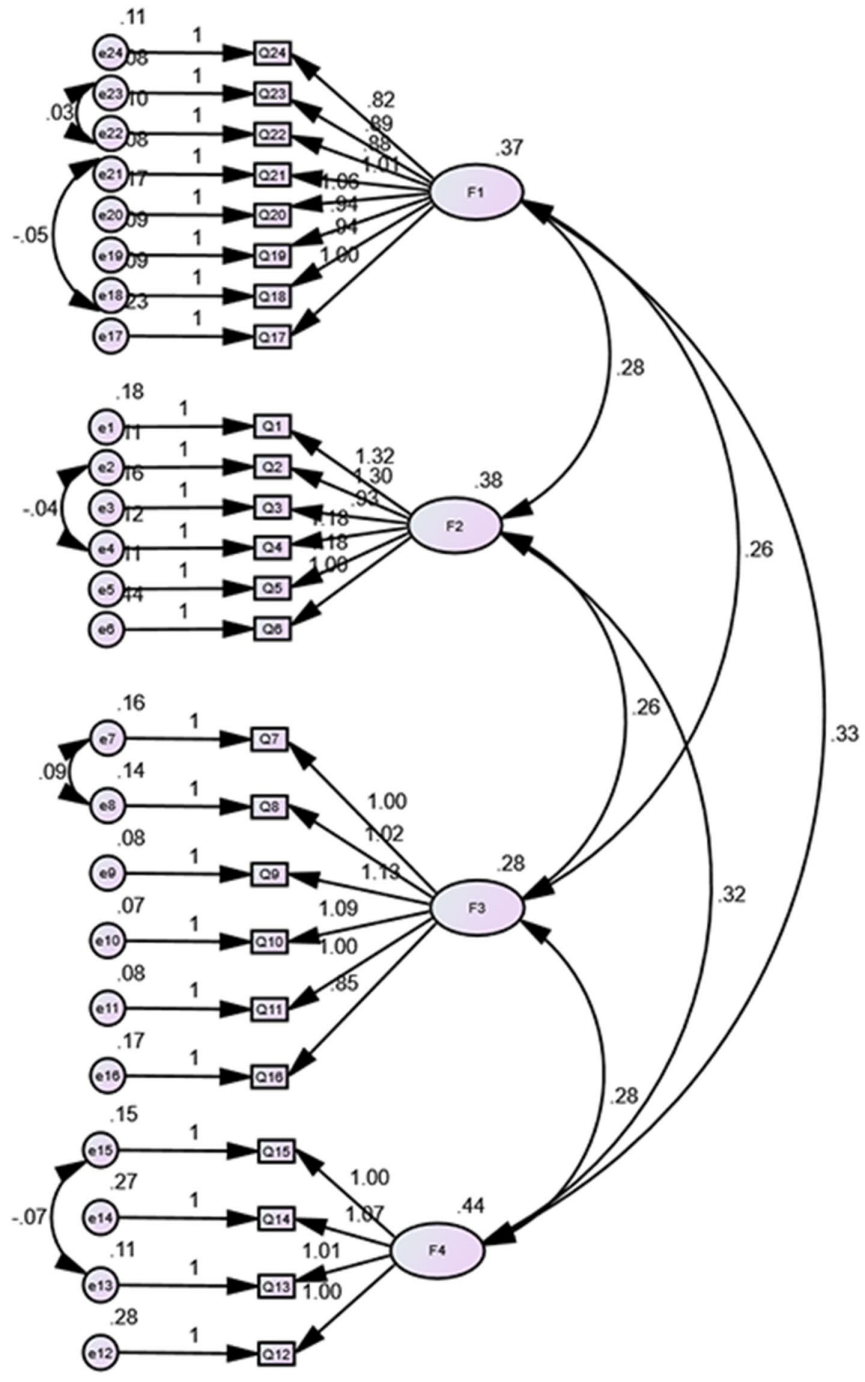
Standardized four-factor structural model of the Chinese version of the IHTSS (*n* = 282)

The results of the CFA are presented in Table 7; Fig. 1. The initial model was iteratively modified based on the modification indices (MI). The adjusted model showed improved fit indices compared to the original model. The CMIN/DF decreased to 2.950 from 3.764, indicating a better fit. The comparative fit index (CFI) increased to 0.935 from 0.906, the incremental fit index (IFI) increased to 0.935 from 0.907, and the goodness-of-fit index (GFI) increased to 0.834 from 0.786.

**Table 7:** Evaluation fitness of IHTSS model

Model	CMIN/DF	*RMSEA*	*RMR*	*CFI*	*GFI*	*IFI*
Initial	3.764	0.101	0.023	0.906	0.786	0.907
Modified model	2.950	0.079	0.023	0.935	0.834	0.935
Standard value	$$ <$$5.000	$$ <$$0.100	$$ <0.05$$0	$$>0.9$$00	$$ >0.$$900	$$ >0.9$$00

### Reliability analysis

The Cronbach’s α value for the Chinese version of IHTSS was found to be 0.970, and split-half reliability to be 0.906, indicating high internal consistency. The four factors within the scale showed Cronbach’s α values ranging from 0.890 to 0.954, suggesting good reliability. To assess the test-retest reliability, the scale was administered again to 30 nurses after a two-week interval, resulting in a reliability coefficient of 0.856. This indicates satisfactory stability and consistency of the scale over time.

## Discussion

The findings reveal that the Chinese version of the IHTSS scale demonstrates excellent psychometric properties, establishing it as an effective and reliable tool to assess the level of intrahospital transport safety for critically ill patients in China. Adhering to the conventions of Chinese expression, slight modifications and translations were made to the original scale using the Brislin translation principles [33]. Simultaneously, multiple nursing experts [34] were involved in this process to ensure compatibility with Chinese idioms and expression patterns. Consistent with previous research, the scale was translated and cross-culturally adapted according to international guidelines. Finally, the Chinese version of the IHTSS exhibited strong internal consistency and suitable use in the ICU setting of China.

In the process of introducing the IHTSS to the Chinese culture, as we all know, not all scales can be easily translated. This study encountered some challenges, given the heterogeneity of cultures. During the EFA processes, four factors were derived, compared to the original five factors scale. The Chinese version of the IHTSS exhibited differences in the classification of teamwork and transport-related tasks compared to the original scale. Certain items, such as item 24 (“I felt supported by the other team members.“), could arguably fall under both categories. Upon thorough examination, it became evident that tasks associated with transport could also be considered part of teamwork. Consequently, the decision to merge these categories was deemed acceptable and comprehensible. Additionally, we made minor adjustments to item 16. item 16 (“We were able to maintain the patient’s privacy during the transport.“) was reclassified from the environmental dimension to the tools and technology dimension. This decision was made after careful consideration of the item’s content, considering the cultural differences that attribute patient privacy exposed to the inappropriate use of nursing tools. The research team extensively discussed the dimensions and agreed that the teamwork and transport-related task dimensions should be renamed as “transport-related tasks and collaboration.” These modifications are not uncommon in the process of translating and culturally adapting scales [31, 35]. The researchers concluded that the disparities between translated scales and the original text depend on various factors, such as the conceptual framework of the study, the evolution of translation methods, language usage, and cultural disparities [36]. These examples highlight the importance of considering cultural factors when translating and adapting scales.

This study demonstrated that the Chinese version of the IHTSS is a reliable and valid assessment instrument. We found that internal consistent reliability, construct validity, content validity, and convergent validity were all acceptable. Our finding of an internal consistency of 0.0.970 is higher than the findings of a Sweden study [26], which found an internal consistency of 0.880, which is deemed very good [37]. The item analysis indicates significant differences in scores between the high and low-scoring groups across all items. This finding suggests that the Chinese version of the IHTSS effectively discriminates between various levels of intrahospital transport safety within ICU settings. Additionally, a notable positive correlation is observed between individual item scores and the total score. These correlations all attain a significant level, providing further confirmation that the Chinese version of the IHTSS satisfies the necessary measurement requirements [38] and should be retained in its entirety. For validity testing, this study examined the validity of the Chinese version of the IHTSS through both structural and content validity. The results of the adjusted model fit indicated positive outcomes, thereby affirming the validity of the translated scale. Previous research found that a scale translated and validated in another sociocultural context with a good fit did not require any changes, aligning with the results of this study [39, 40].

As is the case with most developing countries, China’s critical care nursing rapidly developing, and the safety of critically ill patients is a top priority. The safety management is the key point of intrahospital transport, which is related to the outcome of each critically ill patient and the satisfaction of their families. The application of IHTSS provides a unified and validated tool or provides baseline data about intrahospital transfer safety between different ICUs and different countries.

### Limitations

Due to time and resource constraints, this study focused solely on nurses employed in the tertiary-level ICUs of Hunan Province, thereby excluding other individuals involved in the intrahospital transport of critically ill patients. Consequently, the sample may not be fully representative of the entire population. Future research should strive to enhance the study’s generalizability by expanding the sample size and incorporating participants from hospitals of varying levels and diverse regions, encompassing a wider range of demographic characteristics. Furthermore, it is vital to investigate other groups engaged in the intrahospital transport of critically ill patients to bolster the persuasiveness and relevance of the research findings.

## Conclusion

This study involved the translation and cultural adaptation of the IHTSS into Chinese, followed by an examination of its psychometric properties. The results demonstrated that the Chinese version of the IHTSS exhibits good reliability and validity. The scale’s simplicity in content and structure renders it suitable for measuring the level of intrahospital transport safety for critically ill patients. In the context of rapid advancements in critical care medicine and the ever-changing healthcare environment, this scale provides an effective measurement tool for assessing and improving the intrahospital transport safety of critically ill patients in China. It also serves as a foundation for future intervention research aimed at enhancing the safety of intrahospital transport for critically ill patients.

## Data Availability

The raw data and analysis files supporting the conclusions of this article can be made available from the corresponding author upon reasonable request.

## Abbreviations

IHTSS: The Intrahospital Transport Safety Scale
ICU: intensive care unit
RCIU: Respiratory care unit
General ICU: General intensive care unit
CCU: Coronary Care Unit
EICU: Emergency Intensive Care Unit
NICU: Neurosurgical intensive care unit
SICU: Surgical intensive care unit
CMIN/DF: chi-square/degree of freedom
RMSEA: Root mean square error of Approximation
RMR: Root mean square residual
CFI: Comparative fit index
GFI: Goodness-Of-Fit Index
IFI: Incremental fit index
S-CVI: Scale-level content validity index
I-CVI: Item-level content validity index
KMO: The Kaiser–Meyer–Olkin
EFA: exploratory factor analysis
CFA: Confirmatory factor analysis
MI: The modification indices
CR: Critical ration
